# Baseline survey of the anatomical microbial ecology of an important food plant: *Solanum lycopersicum* (tomato)

**DOI:** 10.1186/1471-2180-13-114

**Published:** 2013-05-24

**Authors:** Andrea R Ottesen, Antonio González Peña, James R White, James B Pettengill, Cong Li, Sarah Allard, Steven Rideout, Marc Allard, Thomas Hill, Peter Evans, Errol Strain, Steven Musser, Rob Knight, Eric Brown

**Affiliations:** 1 FDA Center for Food Safety and Applied Nutrition, Division of Microbiology, Molecular Methods and Subtyping, 5100 Paint Branch Parkway, College Park, MD 20740, USA; 2 Department of Chemistry and Biochemistry, University of Colorado at Boulder 215 UC, Boulder, CO 80309-0215, USA; 3 IGS Institute for Genome Sciences University of Maryland School of Medicine, 801 West Baltimore St., Baltimore, MD 21201, USA; 4 Virginia Polytechnic Institute and State University Eastern Shore AREC, 33446 Research Drive, Painter, VA 23420, USA

**Keywords:** Tomato microflora, 16S, 18S, Metagenomics, Phyllosphere, *Solanum lycopersicum*, Tomato organs, Microbial ecology, Baseline microflora, Tomatome

## Abstract

**Background:**

Research to understand and control microbiological risks associated with the consumption of fresh fruits and vegetables has examined many environments in the farm to fork continuum. An important data gap however, that remains poorly studied is the baseline description of microflora that may be associated with plant anatomy either endemically or in response to environmental pressures. Specific anatomical niches of plants may contribute to persistence of human pathogens in agricultural environments in ways we have yet to describe. Tomatoes have been implicated in outbreaks of *Salmonella* at least 17 times during the years spanning 1990 to 2010. Our research seeks to provide a baseline description of the tomato microbiome and possibly identify whether or not there is something distinctive about tomatoes or their growing ecology that contributes to persistence of *Salmonella* in this important food crop.

**Results:**

DNA was recovered from washes of epiphytic surfaces of tomato anatomical organs; leaves, stems, roots, flowers and fruits of *Solanum lycopersicum* (BHN602), grown at a site in close proximity to commercial farms previously implicated in tomato-*Salmonella* outbreaks. DNA was amplified for targeted 16S and 18S rRNA genes and sheared for shotgun metagenomic sequencing. Amplicons and metagenomes were used to describe “native” bacterial microflora for diverse anatomical parts of Virginia-grown tomatoes.

**Conclusions:**

Distinct groupings of microbial communities were associated with different tomato plant organs and a gradient of compositional similarity could be correlated to the distance of a given plant part from the soil. Unique bacterial phylotypes (at 95% identity) were associated with fruits and flowers of tomato plants. These include *Microvirga, Pseudomonas*, S*phingomonas*, *Brachybacterium*, Rhizobiales, *Paracocccus, Chryseomonas* and *Microbacterium.* The most frequently observed bacterial taxa across aerial plant regions were *Pseudomonas* and *Xanthomonas.* Dominant fungal taxa that could be identified to genus with 18S amplicons included *Hypocrea, Aureobasidium and Cryptococcus.* No definitive presence of *Salmonella* could be confirmed in any of the plant samples, although 16S sequences suggested that closely related genera were present on leaves, fruits and roots.

## Background

The microbial ecology of pathogenicity remains poorly understood in the transmission of many infectious diseases - some of which are vectored by foods. Tomatoes, for example, have been implicated in *Salmonella* outbreaks at least seventeen times in the period spanning 1990 to 2010 (Table 1). Whether or not there are distinctive attributes of tomato plant anatomy or tomato crop field ecology that influence downstream persistence of *Salmonella* in foods remains to be shown.

**Table 1:** ***Salmonella *– Tomato outbreaks**

**Tomato type**	**Outbreak year**	**Location by state**	**Illnesses reported**	***Salmonella *subtype**
Tomato	1990	SC	176	*S*. Javiana
Tomato	1993	SC	100	*S*. Montevideo
Tomato	1998-99	FL	86	*S*. Baildon
Tomato	2000	FL, GA	29	*S*. Thompson
Red Round	2002	VA	512	*S*. Newport
Grape	2002	FL or Mexico	12	*S*. Newport
Roma	2002	FL or Mexico	90	*S*. Javiana
Roma	2004	FL, GA or SC	471	*S*. Javiana
Roma	2004	FL	123	*S*. Braenderup
Red Round	2005	VA	71	*S*. Newport
Tomato	2005	CA	77	*S*. Enteritidis
Roma	2005	FL	76	*S*. Braenderup
Red Round	2006	OH	186	*S*. Typhimurium
Red Round	2006	NA	107	*S*. Newport
Red Round	2007	VA	65	*S*. Newport
Red Round	2010	FL	46	*S*. Newport
Red Round	2010	VA	99	*S*. Newport

Internal FDA list compiled by Captain Thomas Hill.

By the time a fresh fruit or vegetable makes it to the point of human consumption, it has traveled through multiple diverse, yet interwoven, ecologies. It has been affected by agricultural practices, geographic pressures, processing effluents, and microbial landscapes that contribute a vast array of genetic potential. Pathogen-contaminated foods still result in human deaths: as was highlighted in Germany with the *E. coli* O104 outbreak of the summer of 2011 [1]. Since fresh produce is prepared and consumed, often without heating or other types of “kill” steps, a comprehensive understanding of biological risks will improve future risk management.

The number of recognized microbial communities associated with human and environmental ecologies has increased dramatically in the past ten years. A potential “core” microbiome or “enterotypes” of human gut flora have been proposed [2]. Plants, like humans, are comprised of differentiated cells that comprise organs. Microbial constituents of human organs such as skin have been shown to be niche-driven and unique in comparison to one another [3]. It is also likely that different levels of food safety risk correlate with different plant parts, different plant species and the diverse geographic regions in which crops are grown. As we describe the potentially unique “core” microbiomes of human organs – a useful complement for public health research is the study of “core” microbiomes associated with foods. Food microflora intersects with human microflora and influences both health and disease.

Despite an emphasis on “purity” in the Pure Food and Drugs Act of 1906 that largely excludes microbes, it is now understood that almost every food (except, potentially highly processed foods) has a bacterial, fungal, viral and potentially archaeal component to its “naive” (pure) state. The convenience and affordability of next generation sequencing technologies, improved bioinformatic pipelines, and converging reference databases has enabled the description of culture independent microflora associated with numerous environmental and human microbiomes [3-5]. Healthy and diseased states [6] can be correlated to distinctive features of human microbiomes. The networking of interactions among microbiomes of humans, food plants, and agricultural reservoirs will assist epidemiological source tracking of foodborne illnesses. Research into the microbiology of specific points on the farm to consumer continuum has already provided useful information towards minimizing the risks associated with fresh produce [7-9]. Our current study of the epiphytic tomato microbiome (tomatome) addresses one of the many data gaps associated with baseline microbial ecology of food plants.

## Methods

### Field collection of tomato plant parts

Tomato plant parts and fruit (cultivar BHN 602) were collected from research fields at the Virginia Tech Agriculture Research and Education Center in Painter, Virginia (Latitude 37.58, Longitude −75.78). This cultivar shares resistance to specific fungal, bacterial, nematode and viral pressures with other BHN varieties (Additional file 1: Table S1), which accounts for the popularity of BHN tomatoes among commercial growers throughout the eastern United States. Seedlings were started in the green house on 4/29/11 and moved to the field on 6/3/2011. Plants were irrigated using drip tape buried one inch beneath soil level on beds covered with polyethylene mulch. The plots were irrigated daily according to watering needs. Insect, weed control and fertilization was accomplished following the recommendations of the Virginia Cooperative Extension. On July 20th, 2011, four individual plants were taken from four alternating rows, across approximately 30 sq meters of tomato field. At harvest, fruits were mature - predominantly green and breakers (commercial tomatoes in this region are harvested when green). Wearing gloves and using clippers, researchers collected approximately 4 to 6 leaves from both the top third or bottom third of each selected plant; these materials were placed in ziplock bags and considered “Top” and “Bottom” leaf samples respectively. Stems were cut at branching points (6 to 10 per replicate) and six to ten flower cymes were collected per replicate. Fruits (4 per replicate) were taken from various locations on the plants. Roots were unearthed, shaken vigorously, and then cut from the main stem and placed in ziplock bags. All samples were transported back to the lab at ambient temperature and refrigerated at 4 degrees Celsius for 24 hours prior to DNA extraction.

### Nucleic acid extraction

Three hundred milliliters of sterile distilled water were added to each ziplocked bag of plant parts and samples, which was sonicated for 6 minutes to disrupt cells and knock organisms from biofilms or other protective habitat associated with plant organs. This wash was centrifuged and DNA was extracted from the resulting pellet using the Promega Wizard® Genomic DNA purification Kit (Cat.# A1120) (Promega Corporation, Madison, WI) following the extraction protocol for Gram-positive bacterial species.

### 16S rRNA gene amplicon preparation

PCR products designed to target the V2 region of 16S rRNA genes were amplified for Roche pyrosequencing (454) using Roche Fusion Primer A, key (TCAG), and MIDs (Multiplex identifiers for 24 individual samples) and the 27F universal primer: 5’ CGT ATC GCC TCC CTC GCG CCATCAGAGA GTT TGA TCC TGG CTC AG 3’ Reverse primer 533R was used with Roche Fusion Primer B, key, and no mids: 5’ CTA TGC GCC TTG CCA GCC CGC TCAG CGA GAG ATA C TTA CCG CGG CTG CTG GCA C 3’ PCR fragments were cleaned (fragments under 300 bases were removed) using AMPure XP from Beckman Coulter Genomics (Danvers, Massachusetts) at a ratio of 60 μl of AMPure beads to 100 μl PCR product. Remaining PCR fragments were run on the Agilent Bioanalyzer 2100, using the High Sensitivity lab-on-a-chip Reagents (Agilent Technologies, Inc., Santa Clara, CA) to ensure that smaller fragments had been removed prior to emulsion PCR preparation.

### 18S rRNA gene amplicon preparation

EF4 5’GGAAGGGRTGTATTTATTAG 3’ and Fung5 5’GTAAAAGTCCTGGT TCCCC 3’ [10] with 24 MIDs and Roche Fusion Primer adaptors A and B. PCR fragments were cleaned (removal of fragments under 300 bases) using AMPure XP at a ratio of 60 μl of AMPure beads to 100 μl PCR product. Resulting PCR fragments were run on the Bioanalyzer 2100 using to ensure that smaller fragments had been removed prior to emulsion PCR preparation.

### Metagenome preparation

Four independent replicates from each plant organ were pooled to create one representative metagenome for each of the 6 regions: Top Leaves, Flowers, Fruits, Stems, Bottom Leaves, and Roots. DNA was sheared using the Covaris S2 (Woburn, Massachusetts) set for 200 cycles per burst, Duty cycle= 5%, Intensity= 3, for a total of 80 seconds.

### Emulsion PCR

To allow optimal amplification in emulsion, 16S and 18S rRNA gene amplicons were diluted to estimate .3 copies of DNA per bead. Sheared whole genome shotgun (WGS) DNA for metagenomes was diluted to estimate between 3 and 9 copies per bead. Emulsion PCR and breaking and enriching was performed using the Lib-A MV kit for FLX Titanium pyrosequencing from Roche Diagnostics Corp. (Indianapolis, IN) according to the manufacturer’s specifications. For metagenomes, the Lib – L Rapid Library Kit for FLX Titanium pyrosequencing was used according to the manufacturer’s specifications.

### Pyrosequencing

Roche 454 Titanium FLX Approximately 790,000 DNA-enriched beads were loaded into each of 7 quarter regions of two GS Titanium FLX pico titer plates (two separate runs) for sequencing of amplicons and WGS DNA on the Roche 454 GS Titanium FLX platform according to the manufacturer’s specifications.

### Sequence pre-processing

Sequences were processed and split by multiplex identifiers (MIDs) using the sff tools from Roche 454 of Roche Diagnostics Corp. (Indianapolis, IN). Fusion primer sequences detected on the 5’ and 3’ end of sequences were trimmed.

### Bioinformatic analyses: 16S rRNA gene analyses

The Data Intensive Academic Grid (DIAG) computational cloud (http://diagcomputing.org) was used in combination with the CloVR-16S automated pipeline (Version1.1) [11] to perform computationally-intensive tasks, such as chimera detection and nonparametric statistical analyses, on the 16S rRNA gene sequences. The CloVR-16S pipeline utilizes tools for phylogenetic analysis of 16S rRNA data from Qiime [12] and Mothur [13] for sequence processing and diversity analysis, the RDP Bayesian classifier [14] for taxonomic assignment, UCHIME [15] for chimera detection and removal, Metastats [7] for statistical comparisons of sample groups, and various R programs for visualization and unsupervised clustering. A full description of the CloVR-16S standard operating procedure (SOP) is available online at http://clovr.org.

### Phylogenetic analyses of putative Salmonella 16S rRNA gene sequences

We used the approximately-maximum-likelihood method for phylogenetic inference implemented in FastTree [16] to further explore the taxonomic identity of Enterobacteriaceae sequences from the different regions of tomato plants. Reference sequences from Enterobacteriaceae and other phyla observed in the samples were used with *Salmonella* reference sequences from NCBI (Additional file 2: Table S2). Inference was performed using the default settings. Clustering of individuals using the program STRUCTURE [17,18] was performed with K = 2, and K = 3.

### Bioinformatic analyses: 18S rRNA gene analysis

Sequences were clustered stringently using the Qiime UCLUST module set for a 99% identity threshold. Representatives of each cluster (*i.e*., the longest read in each cluster) were examined for chimeras using UCHIME [15] in de novo mode. Clusters identified as chimeras were removed from further analysis. Remaining representatives were searched against the SILVA rRNA small subunit (SSU) [19] database (limited to reference sequences with full taxonomic identification) with BLASTN and a minimum e-value threshold of 1e-5. To provide information about overall fungal distribution, the closest known neighbor for each 99% identity cluster was assigned to the taxonomy of the best-BLAST-hit to the representative sequence.

### Metagenomic analyses

Whole genome shotgun (WGS) metagenomic sequences were provided as input to the CloVR-Metagenomics pipeline (version 1.0) using the “no - Open Read Frameorfs” (no-ORFs) option and the MgRast metagenomics analysis server (version 3.2 Argonne National Laboratory. Argonne, IL http://metagenomics.anl.gov) [20]. Different maximum e-value cutoffs, minimum percentage identity cutoffs and minimum alignment length cutoffs were used for different questions (see individual list in Results section). For overall phylogenetic designation at phylum level – default parameters were 80% similarity over 100 bases at 1e-5. CloVR-Metagenomics was used with a BLAST-based protocol to perform taxonomic and functional annotations as well as statistical analysis with Metastats and R. CloVR pipeline for metagenomes was used with the following SOPs:

1) UCLUST first clusters redundant sequences that show 99% nucleotide identity and removes artificial 454 replicate reads. 2) Representative DNA sequences are searched against the NCBI COG database using BLASTX. 3) Representative DNA sequences are searched against the NCBI RefSeq database of finished prokaryotic genomes using BLASTN. 4) Metastats and CloVR-implemented R scripts are applied for additional statistical and graphical evaluations of the pipeline results. Functional annotation was examined using the COGs database [21]. A full description of the CloVR-Metagenomics SOP is available online at http://clovr.org.

### *Salmonella* detection pipeline

In order to create a pipeline for detecting the presence of *Salmonella*, the IMG contig and genes databases were split into two databases: one that represented all *Salmonella* contigs and genes present in the IMG and the second that represented the remainder of the database (minus all *Salmonella)*. A BLAST approach with extremely relaxed parameters was used to gather hits to *Salmonella* from both of the databases. A bit score with at least 50% the size of the average length of each shotgun data set and a variable id percentage (in this case 40, 50,..100) was used to create plots of hits to *Salmonella* and the bit score of these hits.

### Data Deposition

All metagenomes are available in Mg Rast; accession numbers; 4488526.3 (Bottom Leaves), 4488531.3 (Stems), 4488530.3 (leaves), 4488529.3 (Tomato Fruits), 4488528.3 (Roots), 4488527.3 (Flowers) and SRA at NCBI Genbank (SRA Accession number SRA061333). Submissions conform to the “Minimum Information Standards” [22] recommended by the Genomic Standards Consortium.

## Results and Discussion

**Figure 1:**
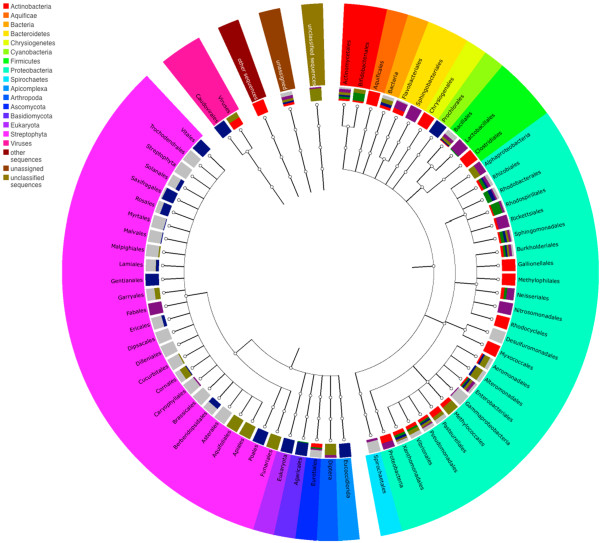
**Phyla associated with tomato anatomy.** Phyla associated with shotgun metagenomic data using M5NR for annotation (Mg Rast version 3.2) with a maximum e-value of 1e-5 and minimum identity of 80%, over 100 bases.

Figure 1 shows ten diverse phyla from bacterial, eukaryotic, and viral domains observed across all the sampled tomato plant organs in the shotgun metagenomic data using M5NR for annotation (Mg Rast version 3.2) with a maximum e-value of 1e-5 and minimum identity of 80%, over 150 bases. A total of 92,695 16S rRNA gene sequences were used to examine bacterial taxonomy and 194,260 18S rRNA gene sequences were used to describe eukaryotes (primarily fungal) associated with diverse tomato organs. In contrast to the other parts of the tomato plants, the most frequently observed bacterial genera from tomato fruit samples were *Pseudomonas, Micrococcineae, Xanthomonas, Methylobacterium, Rhizobium and Sphingomonas.*

**Figure 2:**
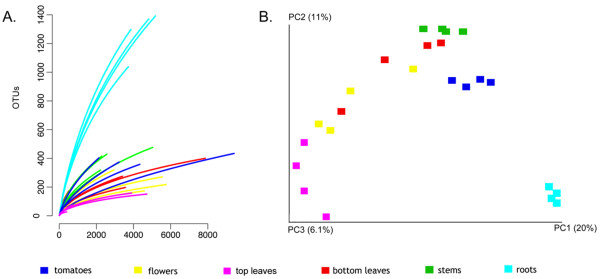
**Number of OTUs per sequences sampled and principal component gradient of unique phylogentic diversity. A.** Rarefaction curves showing diversity of OTUs at 95% associated with tomato organs; roots, leaves (top and bottom), fruits and flowers. **B.** Gradient of unique phylogenetic diversity between bacterial communities associated with each tomato organ.

Rarefaction curves illustrate the number of operational taxonomic units (OTUs) (95%) in relation to sequences sampled for all the plant organs (Figure 2). Not surprisingly, roots have significantly enriched microbial diversity in comparison to all aerial surfaces of the tomato plants. An interesting gradient is observed with regard to the distance of each plant part from the soil: microbial diversity decreases as distance from soil increases (Figure 2).

### Unique and shared bacterial taxa

**Figure 3:**
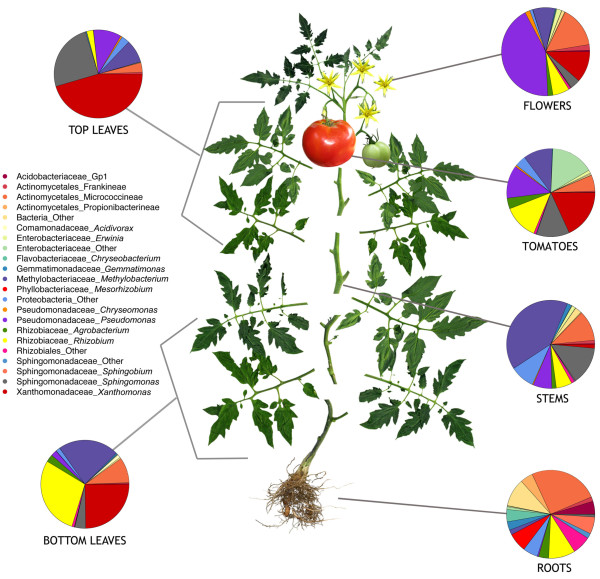
**Bacterial diversity in roots, bottom leaves, stems, tomatoes, flowers and top leaves of tomato plants using 16SrRNA.** Bacterial diversity associated with diverse tomato organs (16S).

Using 95% similarity for selection of OTUs, several OTUs were unique to the combined fruit and flower data sets including; *Microvirga,* Microbacteriaceae, *Sphingomonas, Brachybacterium,* Rhizobiales, *Paracocccus, Chryseomonas* and *Microbacterium.* There were also unique OTUs in root samples, such as *Chryseobacterium, Leifsonia, Pandoraea, Dokdonella, Microbacterium, Arthrobacter, Phyllobacterium, Tetrasphaera, Burkholderia*, and unclassified Intrasporangiaceae. A few bacterial taxa were shared across all 24 independent replicates, including: *Curtobacterium, Methylobacterium, Sphingomonas,* and *Pseudomonas* - suggesting that these taxa may be ubiquitous to the Virginia environment or possibly contaminants from sample preparation. Top bacterial hits by abundance for diverse anatomical regions are shown in Figure 3.

### Fungal elements in tomato microbial ecology

**Figure 4:**
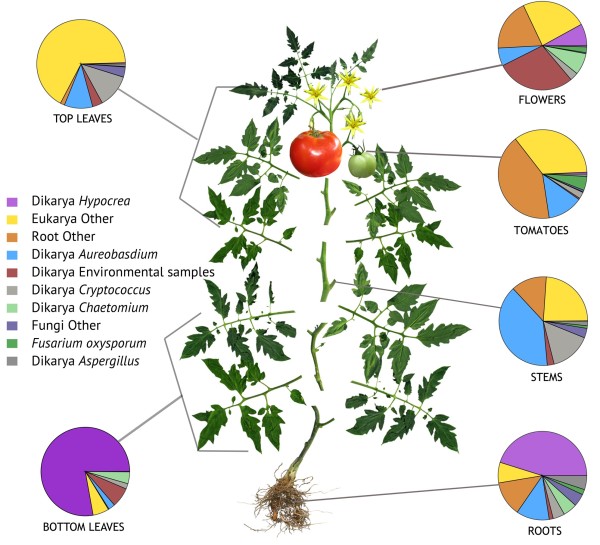
**Fungal diversity in roots, bottom leaves, stems, tomatoes, flowers and top leaves of tomato plants using 18SrRNA.** Fungal diversity associated with diverse tomato organs (18S).

Fungal phyla represented in the 194,260 18S rRNA gene sequences included: Ascomycota, Basidiomycota, Chytridimycota, Glomeromycota, Zygomycota (unclassified) and Mucoromycotina. Dominant fungal genera that could be identified in aerial surfaces were *Hypocrea, Aureobasidium and Cryptococcus* (Figure 4). Three varieties of protists were observed using 18S fungal primers: *Apusomonas*, an endophytic *Actinomycete*, and *Nonomureaea*. Also observed was *Chaetocnema* (flea beetle), a known vector of *Erwinia stewartii*, a close relative of *Salmonella* (alias *Pantoea*), which can result in transmission of Stewart’s wilt, a bacterial wilt of corn.

### Searching for *Salmonella*

**Figure 5:**
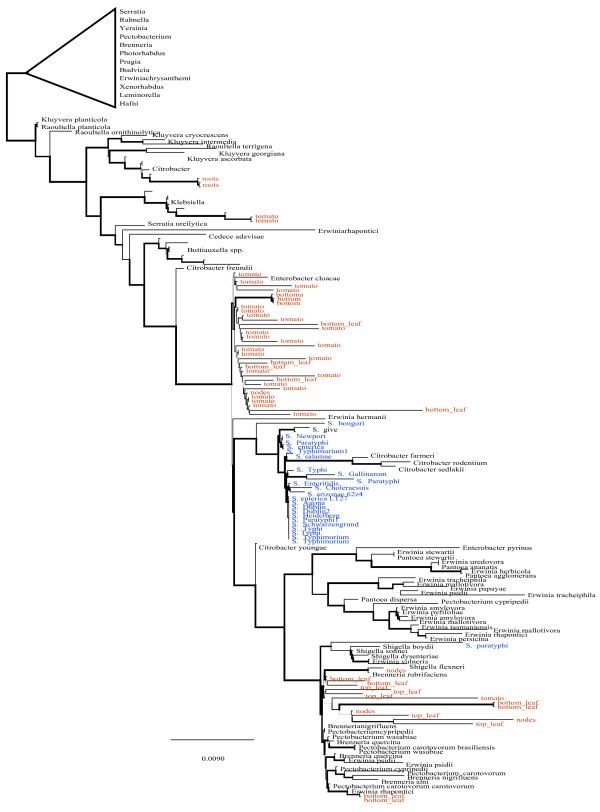
**Tree based examination of *Salmonella *16S sequences.** Phylogenetic placement of putative *Salmonella* 16S rRNA gene sequences from different anatomical regions of tomato plants. Blue sequences are *Salmonella* reference samples (Additional file 2: Table S2) and red sequences are from the tomato anatomy data. A single tip label is used in instances where a clade consists of predominantly one taxa. Phylogenetic placement of putative *Salmonella* 16S rRNA gene sequences from different anatomical regions of tomato plants. Blue sequences are *Salmonella* reference samples (Additional file 2: Table S2) and red sequences are from the tomato anatomy dataset.

**Figure 6:**
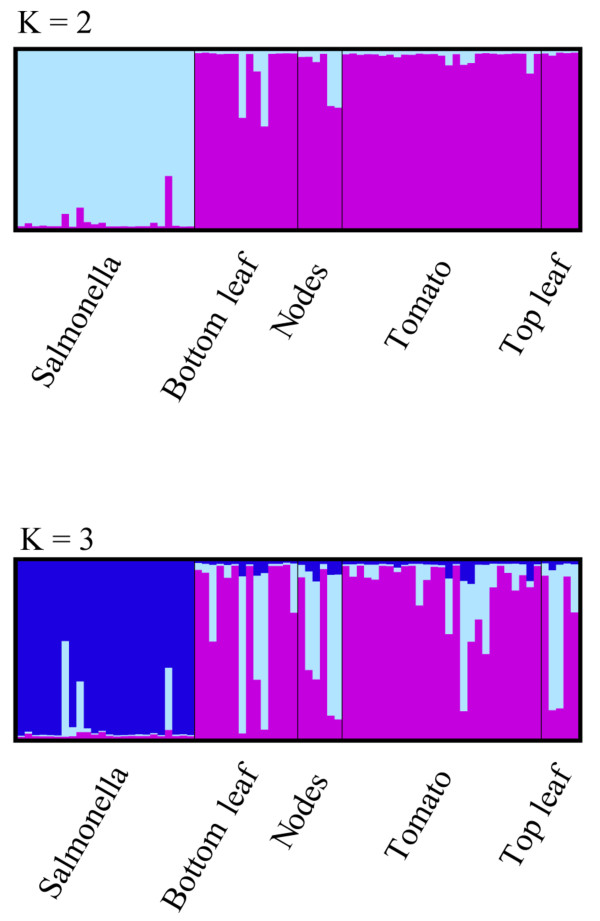
**The clustering of individuals using the program STRUCTURE corroborate the phylogenetic results in that *Salmonella *reference samples are primarily distinct from the isolates identified as being putative *Salmonella *based on BLAST results (Figure**5**).** At K = 2, the reference sequences belong to one cluster and the anatomy samples comprise the second cluster.

Using a cutoff of 97% similarity across 97% of sequence, a few hits to *Salmonella* from the 16S amplicon libraries were identified. Closer phylogenetic inspection (Figures 5 and 6) using tree-based methods with maximum likelihood suggests that the putative *Salmonella* hits were more likely closely related taxa and not in fact*, Salmonella*. Clustering of putative *Salmonella* individuals using the program STRUCTURE corroborated these phylogenetic results and suggested that a representative set of *Salmonella* reference sequences form Genbank belonged to a single cluster and our putative Salmonella sequences from the tomato anatomy samples composed a second cluster (Additional file 2: Table S2). Using the IMG pipeline described in the methods section, no *Salmonella* was detected in any of the shotgun-sequenced metagenomic samples.

### Evolving habitat

The tomato (*Solanum lycopersicum syn. Lycopersicon esculentum)* has been heavily cultivated since the point when it shared a common ancestor with other *Solanum* species such as potato *(Solanum tuberosum),* pepper (*Capsicum sp.,* and eggplant (*Solanum melongena)* some 23 million years ago [23].

Breeding has largely without our noticing, impacted the dynamic interplay of the tomato and its microbial environment for the last 500 years. Quality trait loci (QTL) focused breeding, relying on genomic methods, has drastically sped up the rate of phenotypic change in commercial tomato plants. Thousands of markers across tomato’s 12 chromosomes are correlated to phenotypic characteristics such as thickened pericarps for improved transport durability, joint-less pedicels for ease of processing, ethylene insensitivity for manipulation of ripening dynamics, viral, fungal, nematode and bacterial resistance traits, and many more. While many traits can be mapped to specific chromosomal locations, not even the most experienced of breeders fully understands all the mechanisms in play that contribute to disease resistant phenotypes. Many documented and undocumented phenotypic changes have occurred, and some of these may influence tomato microbial ecology as a reservoir for human pathogens.

For example, epiphytic surfaces of tomato stems, leaves, pedicels and calyxes are covered with at least four different kinds of trichomes, [24] some of which are glandular and emit complex defense chemistries and some of which are smooth and devoid of defense chemistries (Type 1). Work has shown clearly that *Salmonella* preferentially colonizes Type I smooth, long, tomato trichomes [25]. In many commercial cultivars grown today, the number of glandular trichomes and associated defense chemistries have been minimized or lost [26-28]. Perhaps this loss is significant to the composition of microbial communities associated with plant surfaces of *Solanum lycopersicum* cultivars? Whether or not it is important to the flow of pathogens through tomato agriculture remains to be seen. The baseline microbial description presented here for BHN 602 provides information about the microbial communities associated with a heavily bred popular agricultural cultivar of tomato. Future projects that contrast the microbial ecology of commercial cultivars to ancestral varieties would provide an improved understanding of differences that may have occurred in response to an evolving phyllosphere habitat.

Plant organs support a diverse ecological continuum that extends from topical surfaces to endophytic environments. A square centimeter of phyllosphere likely supports anywhere between 10^4^ and 10^9^ cells per cm^2^[29]. Stomata cover the surfaces of tomato plants, even the sepals of the calyx [30]. Epiphytic communities on the exterior of tomato plants play a role in the seeding of endophytic communities associated with internal cellular and vascular habitats. *Salmonella* internalization has been demonstrated in leaves [11] and in developing fruit tissues in laboratory settings [31]. Many have hypothesized that *Salmonella* enters tomato plants via pistillate surfaces of flowers using type III secretion systems – in the same manner that close relative *Erwinia amylovora* invades apple blossoms. Whether or not *Salmonella* internalization by tomatoes is a significant mode of infection for consumers remains to be determined.

Ecologies that contribute to pathogenicity is a quickly expanding focus in public health, and food safety. Research suggests that boundaries between parasitism and mutualism are not as strictly defined as previously believed. Many organisms occupy ecological niches that can shift from pathogenic to symbiotic in response to temporal, genetic, or environmental factors [32]. Certain strains of *Verticillium dahliae* for example, an organism that causes devastating wilts in tomato plants, have been shown to protect tomato plants from more destructive pathovars of *Verticillium* when introduced pre-infection [33].

This paradigm shift supports the need for increased understanding of baseline microbiology associated with foods – especially foods with a history of vectoring disease. Our description of the complex consortia of microbes associated with anatomical organs of *Solanum lycopersicum* provides an interesting baseline for Virginia grown tomatoes that can be used to improve risk assessments for this crop. Future analyses with additional bio-geographical data sets of *Solanum lycopersicum* microflora will help to identify whether or not a “core” microbiome can be ascribed to tomato and if native flora serve as point source contamination or in an ecologically supportive capacity in the flow of pathogens through an agricultural environment.

## Conclusions

**Figure 7:**
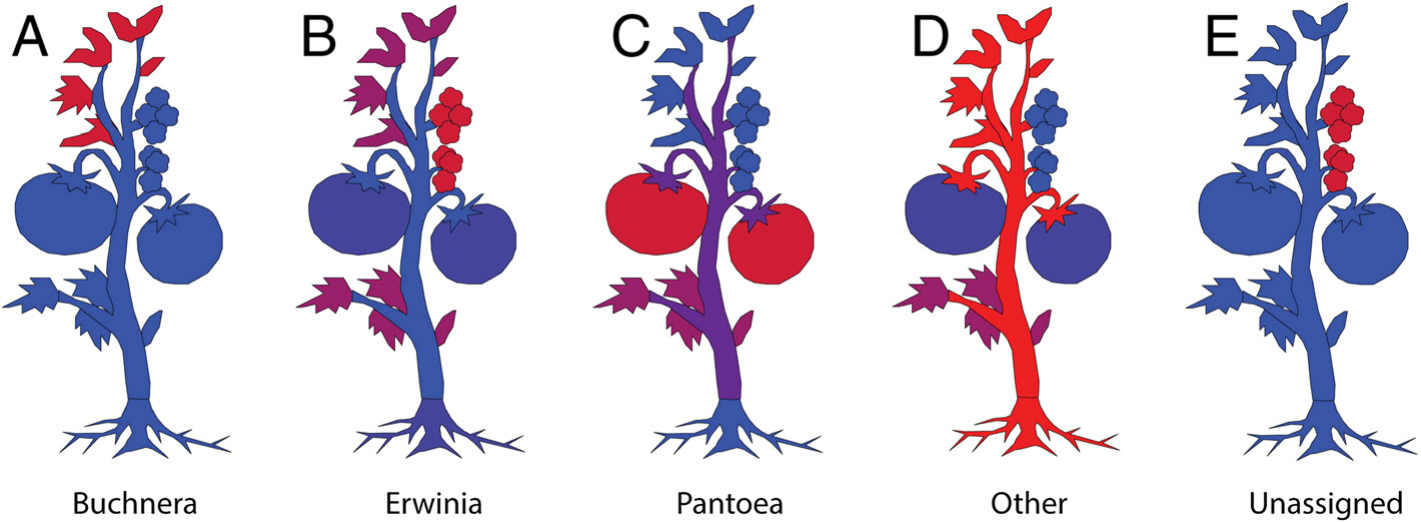
**Taxonomic distribution of representative genera on the tomato plant using 16S with SitePainter.** Images display the geographical location of observed genera (**A**) *Buchnera*, (**B**) *Erwinia*, (**C**) *Pantoea*, (**D**) Other and (**E**) Unassigned, on tomato plants. The sites are colored by abundance, where red represents high abundance, blue represents low abundance and purple represents medium range. The graphic was generated using 16S sequences with SitePainter [34].

It was interesting to observe that distinct groupings and taxa could be ascribed to specific tomato plant organs (Figure 7), while at the same time, a gradient of compositional similarity was correlated to the distance of each plant part from the soil (Figure 2). The latter observation suggests that the observed microflora was influenced by the environment, while the phenomenon of anatomically distinct taxa suggests that the plant niches themselves may be important drivers of microbial community composition. Future work with increased sample sizes and expanded biogeographical regions will help provide higher resolution answers to which influences are most significant to tomato microbial ecology.

## Authors’ contributions

ARO conceived of the study, carried out field and molecular biology sample preparation and drafted the manuscript, AGP, JRW, JPB, RK, and ES performed and advised on bioinformatic analyses, CL assisted with sequencing, SA assisted with field work, SR directed tomato field management, TH advised on tomato-S*almonella* outbreaks, MA, PE, SM, EB supported the work with funding and advisement. All authors read and approved the final manuscript.

## Supplementary Material

Additional file 1: Table S1 BHN resistance *BHN website (*http://www.bhnseed.com/*).* Click here for file

Additional file 2: Table S2 List of Reference *Salmonella* strains used for phylogenetic comparison in **Figure **5. Click here for file
