# Supplementary material for: Baseline survey of the anatomical microbial ecology of an important food plant: Solanum lycopersicum (tomato)
Source: BMC Microbiol. 2013 May 24;13:114. doi: 10.1186/1471-2180-13-114 (PMC3680157; doi:10.1186/1471-2180-13-114)
Supplement: Additional file 1: Table S1 — BHN resistance BHN website (http://www.bhnseed.com/). [file 1471-2180-13-114-S1.docx]

**Supplementary Table 1. BHN resistance**

| **FUNGAL** |
| --- |
| *Verticillium dahliae* |
| *Fusarium oxysporum f. sp. Lycopersici* |
| *Fusarium oxysporum f. sp. radicis-lycopersici* |
| *Alternaria alternata f. sp. Lycopersici* |
| *Alternaria solani* |
| *Stemphylium spp.* |
|  |
| **BACTERIAL** |
| *Pseudomonas syringae* pv. *tomato* |
| *Xanthomonas campestris pv. vesicatoria* |
| *Clavibacter michiganensis* |
| *Ralstonia solanacearum* |
|  |
| **METAZOAL** |
| *Meloidogyne incognita* (Root Knot Nematode) |
|  |
| **VIRAL** |
| Tomato Spotted Wilt Virus |
| Tomato Mosaic Virus |

BHN website (<http://www.bhnseed.com/>)
