# Supplementary figures and images for: Baseline survey of the anatomical microbial ecology of an important food plant: Solanum lycopersicum (tomato)
Source: BMC Microbiol. 2013 May 24;13:114. doi: 10.1186/1471-2180-13-114 (PMC3680157; doi:10.1186/1471-2180-13-114)

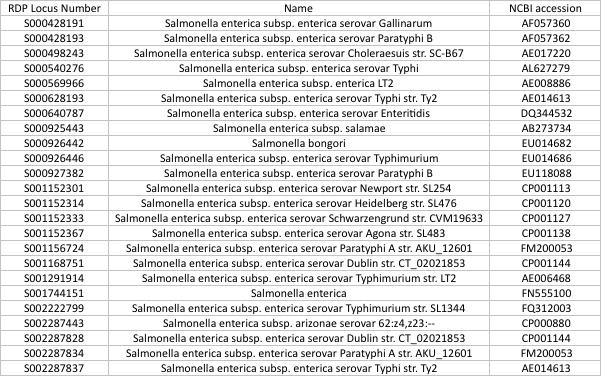

Supplement: Additional file 2: Table S2 — List of Reference Salmonella strains used for phylogenetic comparison in Figure 5. [file 1471-2180-13-114-S2.docx]
